# (2-Meth­oxy­benz­yl)(2-meth­oxy­benzyl­idene)aza­nium (2-meth­oxy­phen­yl)methanaminium tetra­chloridozincate(II) monohydrate

**DOI:** 10.1107/S1600536810021793

**Published:** 2010-06-16

**Authors:** Meher El Glaoui, Erwann Jeanneau, Matthias Zeller, Frederic Lefebvre, Cherif Ben Nasr

**Affiliations:** a Laboratoire de Chimie des Matériaux, Faculté des Sciences de Bizerte, 7021 Zarzouna, Tunisia; b Universtié Lyon1, Centre de Diffractométrie Henri Longchambon, 43 boulevard du 11 Novembre 1918, 69622 Villeurbanne Cedex, France; c Youngstown State University, Department of Chemistry, One University Plaza, Youngstown, Ohio 44555-3663, USA; d Laboratoire de Chimie Organometallique de Surface (LCOMS), Ecole Superieure de Chimie Physique Electronique, 69622 Villeurbanne Cedex, France

## Abstract

The title compound, (C_8_H_12_NO)(C_16_H_18_NO_2_)[ZnCl_4_]·H_2_O, was obtained as a by-product of the Zn^2+^ and HCl catalyzed condensation of (2-meth­oxy­phen­yl)methanamine in water. Both cations feature an intra­molecular N—H⋯O hydrogen bond. In the crystal, the components are linked by an extensive three-dimensional network of N—H⋯O, O—H⋯Cl and N—H⋯Cl hydrogen bonds (three of them bifurcated). Weak C—H⋯O intera­ctions also occur.

## Related literature

For related *meta*-chlorido complexes, see: Ben Gharbia *et al.* (2005 ▶, 2008 ▶). For Zn—Cl distances and Cl—Zn—Cl bond angles, see: Gayathri *et al.* (2008 ▶); Hosseinian & Mahjoub (2009 ▶).
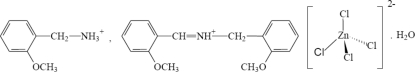


## Experimental

### 

#### Crystal data

(C_8_H_12_NO)(C_16_H_18_NO_2_)[ZnCl_4_]·H_2_O
*M* *_r_* = 619.69
Triclinic, 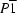

*a* = 8.0884 (9) Å
*b* = 12.424 (2) Å
*c* = 14.678 (2) Å
α = 98.23 (1)°
β = 97.43 (1)°
γ = 90.29 (1)°
*V* = 1447.1 (3) Å^3^
*Z* = 2
Mo *K*α radiation
μ = 1.25 mm^−1^
*T* = 293 K
0.54 × 0.47 × 0.25 mm

#### Data collection

Oxford Diffraction Xcalibur diffractometer
Absorption correction: analytical (*CrysAlis PRO*; Oxford Diffraction, 2009 ▶; Clark & Reid, 1995 ▶) *T*_min_ = 0.576, *T*_max_ = 0.749
12584 measured reflections
6583 independent reflections
4644 reflections with *I* > 2σ(*I*)
*R*_int_ = 0.028

#### Refinement

*R*[*F* ^2^ > 2σ(*F* ^2^)] = 0.036
*wR*(*F* ^2^) = 0.087
*S* = 0.98
6583 reflections
329 parameters
3 restraints
H atoms treated by a mixture of independent and constrained refinement
Δρ_max_ = 0.72 e Å^−3^
Δρ_min_ = −0.64 e Å^−3^

### 

Data collection: *CrysAlis PRO* (Oxford Diffraction, 2009 ▶); cell refinement: *CrysAlis PRO*; data reduction: *CrysAlis PRO*; program(s) used to solve structure: *SIR97* (Altomare *et al.*, 1999 ▶); program(s) used to refine structure: *SHELXTL* (Sheldrick, 2008 ▶); molecular graphics: *SHELXTL*; software used to prepare material for publication: *SHELXTL*.

## Supplementary Material

Crystal structure: contains datablocks global, I. DOI: 10.1107/S1600536810021793/hb5489sup1.cif

Structure factors: contains datablocks I. DOI: 10.1107/S1600536810021793/hb5489Isup2.hkl

Additional supplementary materials:  crystallographic information; 3D view; checkCIF report

## Figures and Tables

**Table 1:** Hydrogen-bond geometry (Å, °)

*D*—H⋯*A*	*D*—H	H⋯*A*	*D*⋯*A*	*D*—H⋯*A*
N1—H1⋯O2	0.89 (1)	2.07 (2)	2.680 (2)	125 (2)
N1—H1⋯Cl2^i^	0.89 (1)	2.64 (2)	3.3221 (18)	135 (2)
N2—H2*A*⋯O4	0.89	2.30	3.102 (3)	151
N2—H2*A*⋯O3	0.89	2.37	2.877 (2)	116
N2—H2*B*⋯O4^ii^	0.89	2.04	2.881 (3)	158
N2—H2*B*⋯Cl3^iii^	0.89	2.98	3.502 (2)	120
N2—H2*C*⋯Cl1^iv^	0.89	2.45	3.306 (2)	162
O4—H4*B*⋯Cl4^iii^	0.80 (2)	2.44 (2)	3.2323 (19)	168 (3)
O4—H4*A*⋯Cl1^v^	0.80 (2)	2.72 (2)	3.4165 (19)	147 (3)
C8—H8*A*⋯Cl3	0.97	2.67	3.474 (2)	140
C11—H11⋯Cl2	0.93	2.82	3.687 (2)	155
C24—H24*A*⋯Cl4^vi^	0.97	2.76	3.707 (3)	166

Symmetry codes: (i) 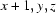; (ii) 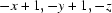; (iii) 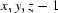; (iv) 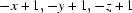; (v) 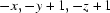; (vi) 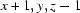.

**Fig. 1.**
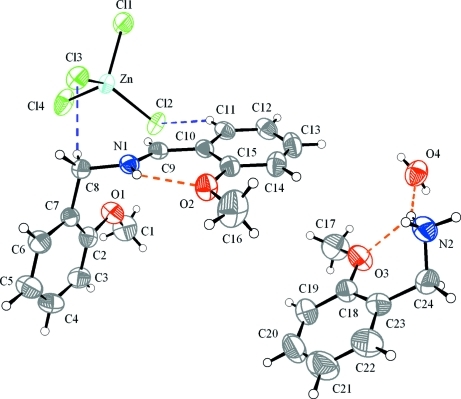
A view of the title compound, showing 50% probability displacement ellipsoids (arbitrary spheres for the H atoms).

**Fig. 2.**
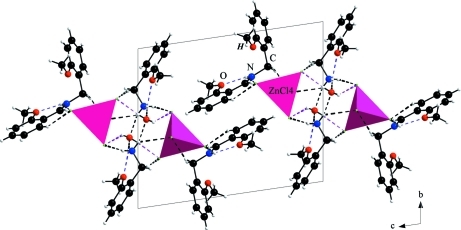
Projection of the structure along the a axis. Hydrogen bonds are denoted by dotted lines.

## supplementary crystallographic information

### Comment

As a part of our ongoing investigations in molecular salts containing *meta*-chlorido complexes (Ben Gharbia *et al.*, 2005; Ben Gharbia *et al.*, 2008), we report here the crystal structure of one such compound, (C_16_H_18_NO_2_)(C_8_H_12_NO)[ZnCl_4_].H_2_O. The title compound was obtained as a byproduct of the Zn^2+^ and HCl catalyzed condensation of (2-methoxyphenyl) methanamine in water. Subsequent crystallization from the reaction mixture yielded among the main reaction products crystals of the title compound that consist of one *N*-(2-methoxybenzylidene)-1-(2-methoxyphenyl)methanaminnium cation, one (2-methoxyphenyl) methanaminium cation, one ZnCl_4_^2-^ anion and one interstitial water molecule (Fig. 1).

The distance N1—C9 [1.273 (2) Å] is substantially shorter than the one of N1—C8 [1.477 (2) Å], indicating the presence of a double bond in the condensation product, thus indicating the nature of the organic molecules in the crystal as indictated in Scheme 1. Preliminary NMR data on the material indicate that the bulk of the reaction product is not identical with the title compound. Further investigations into the nature of the bulk material are under way.

The Cl—Zn—Cl bond angles in the title compound show relatively little distortion from a regular tetrahedron [spread values 104.45 (3)–111.78 (2)] (Gayathri *et al.*, 2008, Hosseinian *et al.*, 2009). Classic N—H···O, O—H···Cl and N—H···Cl hydrogen bonds are observed, which link the two types of organic ammonium cations, the anionic complexes [ZnCl_4_]^2-^ and the uncoordinated water molecules into a 3-D hydrogen bonded network, as shown in Fig. 2. Three of the hydrogen bonds are bifurcated: N1—H1··· (Cl2,O2), N2—H2A···(O3,O4) and N2—H2B···(O4,Cl3).

### Experimental

An aqueous solution of (2-methoxyphenyl) methanamine (2-methoxybenzylamine), zinc chloride and 1 *M* hydrochloric acid in a Petri disk was slowly evaporated at room temperature.  A colourless block of (I), which remained stable under normal conditions of temperature and humidity, was isolated as a byproduct of the reaction.

### Refinement

C—H and ammonium H atoms were placed in calculated positions with C—H in the range 0.93–0.97 and N—H equal to 0.89 Å. The imminium and the water hydrogen atom postitions were refined with N—H and O—H distance restraints of 0.89 (2) and 0.82 (2) Å. The *U*_iso_(H) values of all H atoms were constrained to 1.2 or 1.5 times *U*_eq_ of the respective parent atom.

### Crystal data

**Table:**

(C_8_H_12_NO)(C_16_H_18_NO_2_)[ZnCl_4_]·H_2_O	*Z* = 2
*M**_r_* = 619.69	*F*(000) = 640
Triclinic, *P*1	*D*_x_ = 1.422 Mg m^−^^3^
Hall symbol: -P 1	Mo *K*α radiation, λ = 0.71069 Å
*a* = 8.0884 (9) Å	Cell parameters from 5313 reflections
*b* = 12.424 (2) Å	θ = 3.0–29.2°
*c* = 14.678 (2) Å	µ = 1.25 mm^−^^1^
α = 98.23 (1)°	*T* = 293 K
β = 97.43 (1)°	Block, colourless
γ = 90.29 (1)°	0.54 × 0.47 × 0.25 mm
*V* = 1447.1 (3) Å^3^	

### Data collection

**Table:**

Oxford Diffraction Xcalibur diffractometer	6583 independent reflections
Radiation source: fine-focus sealed tube	4644 reflections with *I* > 2σ(*I*)
graphite	*R*_int_ = 0.028
ω scans	θ_max_ = 29.5°, θ_min_ = 3.0°
Absorption correction: analytical (*CrysAlis PRO*; Oxford Diffraction, 2009; Clark & Reid, 1995)	*h* = −10→11
*T*_min_ = 0.576, *T*_max_ = 0.749	*k* = −15→16
12584 measured reflections	*l* = −19→19

### Refinement

**Table:**

Refinement on *F*^2^	Primary atom site location: structure-invariant direct methods
Least-squares matrix: full	Secondary atom site location: difference Fourier map
*R*[*F*^2^ > 2σ(*F*^2^)] = 0.036	Hydrogen site location: inferred from neighbouring sites
*wR*(*F*^2^) = 0.087	H atoms treated by a mixture of independent and constrained refinement
*S* = 0.98	*w* = 1/[σ^2^(*F*_o_^2^) + (0.0435*P*)^2^] where *P* = (*F*_o_^2^ + 2*F*_c_^2^)/3
6583 reflections	(Δ/σ)_max_ = 0.001
329 parameters	Δρ_max_ = 0.72 e Å^−^^3^
3 restraints	Δρ_min_ = −0.64 e Å^−^^3^

### Special details

**Table:**

Geometry. All e.s.d.'s (except the e.s.d. in the dihedral angle between two l.s. planes) are estimated using the full covariance matrix. The cell e.s.d.'s are taken into account individually in the estimation of e.s.d.'s in distances, angles and torsion angles; correlations between e.s.d.'s in cell parameters are only used when they are defined by crystal symmetry. An approximate (isotropic) treatment of cell e.s.d.'s is used for estimating e.s.d.'s involving l.s. planes.
Refinement. Refinement of *F*^2^ against ALL reflections. The weighted *R*-factor *wR* and goodness of fit *S* are based on *F*^2^, conventional *R*-factors *R* are based on *F*, with *F* set to zero for negative *F*^2^. The threshold expression of *F*^2^ > σ(*F*^2^) is used only for calculating *R*-factors(gt) *etc*. and is not relevant to the choice of reflections for refinement. *R*-factors based on *F*^2^ are statistically about twice as large as those based on *F*, and *R*- factors based on ALL data will be even larger.

### Fractional atomic coordinates and isotropic or equivalent isotropic displacement parameters (Å^2^)

**Table:**

	*x*	*y*	*z*	*U*_iso_*/*U*_eq_	
C1	0.2523 (3)	0.0313 (2)	0.58855 (19)	0.0669 (8)	
H1A	0.2600	−0.0055	0.5272	0.100*	
H1B	0.1579	0.0778	0.5869	0.100*	
H1C	0.2392	−0.0214	0.6292	0.100*	
C2	0.5461 (3)	0.04191 (18)	0.63917 (14)	0.0377 (5)	
C3	0.5657 (3)	−0.06829 (19)	0.61983 (16)	0.0486 (6)	
H3	0.4754	−0.1130	0.5916	0.058*	
C4	0.7185 (3)	−0.1128 (2)	0.64206 (17)	0.0559 (6)	
H4	0.7313	−0.1875	0.6285	0.067*	
C5	0.8521 (3)	−0.0475 (2)	0.68406 (17)	0.0535 (6)	
H5	0.9549	−0.0779	0.6999	0.064*	
C6	0.8333 (3)	0.06297 (19)	0.70261 (14)	0.0421 (5)	
H6	0.9247	0.1070	0.7303	0.051*	
C7	0.6816 (2)	0.11022 (17)	0.68108 (13)	0.0331 (4)	
C8	0.6598 (3)	0.23148 (16)	0.70157 (13)	0.0367 (5)	
H8A	0.5629	0.2461	0.7336	0.044*	
H8B	0.7568	0.2646	0.7418	0.044*	
C9	0.5018 (2)	0.31536 (16)	0.57852 (13)	0.0331 (4)	
H9	0.4129	0.3162	0.6129	0.040*	
C10	0.4693 (2)	0.35513 (16)	0.49025 (13)	0.0326 (4)	
C11	0.3132 (2)	0.39947 (17)	0.46852 (15)	0.0390 (5)	
H11	0.2380	0.4048	0.5117	0.047*	
C12	0.2683 (3)	0.43548 (18)	0.38452 (16)	0.0485 (6)	
H12	0.1641	0.4649	0.3709	0.058*	
C13	0.3800 (3)	0.42721 (19)	0.32143 (16)	0.0536 (6)	
H13	0.3498	0.4506	0.2643	0.064*	
C14	0.5355 (3)	0.3854 (2)	0.34018 (15)	0.0469 (6)	
H14	0.6098	0.3814	0.2965	0.056*	
C15	0.5808 (2)	0.34898 (17)	0.42502 (14)	0.0360 (5)	
C16	0.8537 (3)	0.2994 (3)	0.38782 (19)	0.0764 (9)	
H16A	0.8119	0.2554	0.3301	0.115*	
H16B	0.9527	0.2678	0.4153	0.115*	
H16C	0.8797	0.3714	0.3766	0.115*	
C17	0.2513 (3)	0.1890 (3)	0.1188 (2)	0.0783 (9)	
H17A	0.2140	0.1149	0.0986	0.117*	
H17B	0.1647	0.2370	0.1003	0.117*	
H17C	0.2776	0.2010	0.1852	0.117*	
C18	0.5273 (3)	0.14345 (19)	0.08828 (15)	0.0467 (6)	
C19	0.5371 (4)	0.0578 (2)	0.14046 (17)	0.0616 (7)	
H19	0.4496	0.0425	0.1722	0.074*	
C20	0.6792 (4)	−0.0042 (2)	0.14433 (19)	0.0741 (8)	
H20	0.6864	−0.0621	0.1783	0.089*	
C21	0.8083 (4)	0.0192 (3)	0.0987 (2)	0.0772 (9)	
H21	0.9038	−0.0221	0.1023	0.093*	
C22	0.7973 (3)	0.1043 (2)	0.04706 (19)	0.0641 (7)	
H22	0.8862	0.1200	0.0164	0.077*	
C23	0.6558 (3)	0.16659 (19)	0.04026 (15)	0.0435 (5)	
C24	0.6378 (3)	0.2543 (2)	−0.02067 (16)	0.0512 (6)	
H24A	0.7340	0.2540	−0.0539	0.061*	
H24B	0.5397	0.2379	−0.0663	0.061*	
Cl3	0.40847 (6)	0.41374 (5)	0.81903 (4)	0.04914 (15)	
Cl2	0.04753 (7)	0.32552 (5)	0.63718 (3)	0.05224 (16)	
Cl1	0.03902 (8)	0.57253 (6)	0.81699 (4)	0.06066 (18)	
Cl4	0.03288 (9)	0.29378 (6)	0.88415 (4)	0.0746 (2)	
N1	0.6383 (2)	0.27933 (13)	0.61445 (11)	0.0334 (4)	
H1	0.729 (2)	0.2772 (17)	0.5852 (13)	0.040*	
N2	0.6225 (2)	0.36549 (15)	0.03186 (13)	0.0488 (5)	
H2A	0.5218	0.3717	0.0501	0.073*	
H2B	0.6365	0.4152	−0.0047	0.073*	
H2C	0.7000	0.3756	0.0814	0.073*	
O1	0.40047 (18)	0.09490 (13)	0.62188 (11)	0.0508 (4)	
O2	0.72997 (17)	0.30455 (13)	0.44960 (10)	0.0458 (4)	
O3	0.3943 (2)	0.20972 (14)	0.07819 (12)	0.0616 (5)	
O4	0.2655 (2)	0.45092 (16)	0.04551 (11)	0.0573 (5)	
H4B	0.220 (3)	0.412 (2)	0.0009 (15)	0.086*	
H4A	0.208 (3)	0.473 (3)	0.0836 (17)	0.086*	
Zn1	0.12911 (3)	0.39816 (2)	0.786811 (15)	0.03902 (9)	

### Atomic displacement parameters (Å^2^)

**Table:**

	*U*^11^	*U*^22^	*U*^33^	*U*^12^	*U*^13^	*U*^23^
C1	0.0402 (13)	0.079 (2)	0.0807 (19)	−0.0143 (13)	0.0011 (13)	0.0147 (16)
C2	0.0393 (12)	0.0406 (14)	0.0358 (11)	−0.0040 (10)	0.0081 (9)	0.0113 (10)
C3	0.0548 (14)	0.0362 (14)	0.0524 (14)	−0.0100 (11)	0.0027 (11)	0.0031 (12)
C4	0.0713 (18)	0.0304 (14)	0.0637 (16)	0.0043 (12)	0.0050 (13)	0.0018 (12)
C5	0.0504 (14)	0.0428 (16)	0.0665 (16)	0.0090 (11)	0.0044 (12)	0.0085 (13)
C6	0.0401 (12)	0.0393 (14)	0.0461 (13)	−0.0011 (10)	0.0011 (10)	0.0075 (11)
C7	0.0400 (11)	0.0314 (12)	0.0299 (10)	−0.0007 (9)	0.0076 (8)	0.0080 (9)
C8	0.0462 (12)	0.0327 (12)	0.0311 (10)	0.0012 (9)	0.0046 (9)	0.0050 (9)
C9	0.0349 (11)	0.0276 (11)	0.0367 (11)	−0.0013 (8)	0.0073 (9)	0.0024 (9)
C10	0.0343 (10)	0.0275 (11)	0.0357 (11)	−0.0030 (8)	0.0015 (8)	0.0061 (9)
C11	0.0349 (11)	0.0307 (12)	0.0504 (13)	−0.0035 (9)	0.0017 (10)	0.0061 (10)
C12	0.0416 (12)	0.0413 (14)	0.0599 (15)	−0.0027 (10)	−0.0104 (11)	0.0142 (12)
C13	0.0635 (16)	0.0510 (16)	0.0454 (13)	−0.0112 (12)	−0.0118 (12)	0.0208 (12)
C14	0.0510 (14)	0.0531 (15)	0.0377 (12)	−0.0075 (11)	0.0052 (10)	0.0118 (11)
C15	0.0355 (11)	0.0327 (12)	0.0393 (11)	−0.0066 (9)	0.0008 (9)	0.0073 (10)
C16	0.0522 (16)	0.116 (3)	0.0707 (18)	0.0130 (16)	0.0307 (14)	0.0248 (18)
C17	0.0612 (17)	0.080 (2)	0.104 (2)	0.0048 (15)	0.0403 (17)	0.0217 (19)
C18	0.0518 (14)	0.0454 (15)	0.0433 (13)	0.0018 (11)	0.0046 (11)	0.0090 (12)
C19	0.0748 (18)	0.0559 (18)	0.0588 (16)	0.0021 (14)	0.0137 (14)	0.0202 (14)
C20	0.101 (2)	0.058 (2)	0.0637 (18)	0.0116 (17)	−0.0055 (17)	0.0245 (16)
C21	0.069 (2)	0.075 (2)	0.084 (2)	0.0259 (16)	−0.0060 (16)	0.0126 (18)
C22	0.0508 (15)	0.066 (2)	0.0751 (18)	0.0077 (13)	0.0082 (13)	0.0080 (16)
C23	0.0402 (12)	0.0427 (14)	0.0457 (13)	0.0002 (10)	0.0025 (10)	0.0019 (11)
C24	0.0579 (15)	0.0516 (16)	0.0472 (13)	−0.0012 (12)	0.0177 (11)	0.0080 (12)
Cl3	0.0335 (3)	0.0638 (4)	0.0473 (3)	0.0050 (3)	0.0046 (2)	−0.0010 (3)
Cl2	0.0449 (3)	0.0768 (5)	0.0322 (3)	−0.0055 (3)	0.0033 (2)	0.0003 (3)
Cl1	0.0586 (4)	0.0643 (5)	0.0540 (4)	0.0230 (3)	−0.0034 (3)	0.0003 (3)
Cl4	0.0860 (5)	0.0963 (6)	0.0413 (3)	−0.0483 (4)	0.0023 (3)	0.0165 (4)
N1	0.0369 (9)	0.0298 (10)	0.0347 (9)	−0.0008 (8)	0.0067 (7)	0.0067 (8)
N2	0.0512 (11)	0.0436 (12)	0.0522 (11)	−0.0044 (9)	0.0039 (9)	0.0118 (10)
O1	0.0377 (8)	0.0492 (10)	0.0652 (10)	−0.0031 (7)	0.0023 (7)	0.0120 (8)
O2	0.0374 (8)	0.0595 (11)	0.0444 (8)	0.0057 (7)	0.0109 (7)	0.0159 (8)
O3	0.0493 (10)	0.0612 (12)	0.0850 (13)	0.0119 (8)	0.0259 (9)	0.0309 (10)
O4	0.0568 (11)	0.0688 (13)	0.0442 (10)	−0.0155 (9)	0.0040 (8)	0.0048 (10)
Zn1	0.03518 (14)	0.05046 (18)	0.03133 (14)	−0.00161 (11)	0.00427 (10)	0.00579 (12)

### Geometric parameters (Å, °)

**Table:**

C1—O1	1.424 (3)	C16—O2	1.431 (2)
C1—H1A	0.9600	C16—H16A	0.9600
C1—H1B	0.9600	C16—H16B	0.9600
C1—H1C	0.9600	C16—H16C	0.9600
C2—O1	1.365 (2)	C17—O3	1.407 (3)
C2—C3	1.372 (3)	C17—H17A	0.9600
C2—C7	1.402 (3)	C17—H17B	0.9600
C3—C4	1.377 (3)	C17—H17C	0.9600
C3—H3	0.9300	C18—O3	1.364 (3)
C4—C5	1.372 (3)	C18—C23	1.380 (3)
C4—H4	0.9300	C18—C19	1.394 (3)
C5—C6	1.374 (3)	C19—C20	1.386 (4)
C5—H5	0.9300	C19—H19	0.9300
C6—C7	1.382 (3)	C20—C21	1.362 (4)
C6—H6	0.9300	C20—H20	0.9300
C7—C8	1.509 (3)	C21—C22	1.384 (4)
C8—N1	1.477 (2)	C21—H21	0.9300
C8—H8A	0.9700	C22—C23	1.386 (3)
C8—H8B	0.9700	C22—H22	0.9300
C9—N1	1.273 (2)	C23—C24	1.501 (3)
C9—C10	1.445 (3)	C24—N2	1.497 (3)
C9—H9	0.9300	C24—H24A	0.9700
C10—C15	1.392 (3)	C24—H24B	0.9700
C10—C11	1.397 (3)	Cl3—Zn1	2.2495 (6)
C11—C12	1.377 (3)	Cl2—Zn1	2.2639 (6)
C11—H11	0.9300	Cl1—Zn1	2.2903 (8)
C12—C13	1.369 (3)	Cl4—Zn1	2.2664 (7)
C12—H12	0.9300	N1—H1	0.893 (14)
C13—C14	1.373 (3)	N2—H2A	0.8900
C13—H13	0.9300	N2—H2B	0.8900
C14—C15	1.391 (3)	N2—H2C	0.8900
C14—H14	0.9300	O4—H4B	0.801 (17)
C15—O2	1.357 (2)	O4—H4A	0.795 (17)
			
O1—C1—H1A	109.5	H16A—C16—H16B	109.5
O1—C1—H1B	109.5	O2—C16—H16C	109.5
H1A—C1—H1B	109.5	H16A—C16—H16C	109.5
O1—C1—H1C	109.5	H16B—C16—H16C	109.5
H1A—C1—H1C	109.5	O3—C17—H17A	109.5
H1B—C1—H1C	109.5	O3—C17—H17B	109.5
O1—C2—C3	125.4 (2)	H17A—C17—H17B	109.5
O1—C2—C7	114.2 (2)	O3—C17—H17C	109.5
C3—C2—C7	120.4 (2)	H17A—C17—H17C	109.5
C2—C3—C4	120.2 (2)	H17B—C17—H17C	109.5
C2—C3—H3	119.9	O3—C18—C23	114.11 (19)
C4—C3—H3	119.9	O3—C18—C19	124.9 (2)
C5—C4—C3	120.3 (2)	C23—C18—C19	121.0 (2)
C5—C4—H4	119.9	C20—C19—C18	119.1 (2)
C3—C4—H4	119.9	C20—C19—H19	120.4
C4—C5—C6	119.6 (2)	C18—C19—H19	120.4
C4—C5—H5	120.2	C21—C20—C19	120.4 (3)
C6—C5—H5	120.2	C21—C20—H20	119.8
C5—C6—C7	121.5 (2)	C19—C20—H20	119.8
C5—C6—H6	119.3	C20—C21—C22	120.1 (3)
C7—C6—H6	119.3	C20—C21—H21	120.0
C6—C7—C2	118.0 (2)	C22—C21—H21	120.0
C6—C7—C8	121.79 (19)	C21—C22—C23	121.0 (3)
C2—C7—C8	120.23 (18)	C21—C22—H22	119.5
N1—C8—C7	110.25 (16)	C23—C22—H22	119.5
N1—C8—H8A	109.6	C18—C23—C22	118.4 (2)
C7—C8—H8A	109.6	C18—C23—C24	120.1 (2)
N1—C8—H8B	109.6	C22—C23—C24	121.5 (2)
C7—C8—H8B	109.6	N2—C24—C23	113.39 (18)
H8A—C8—H8B	108.1	N2—C24—H24A	108.9
N1—C9—C10	127.33 (18)	C23—C24—H24A	108.9
N1—C9—H9	116.3	N2—C24—H24B	108.9
C10—C9—H9	116.3	C23—C24—H24B	108.9
C15—C10—C11	118.40 (18)	H24A—C24—H24B	107.7
C15—C10—C9	124.36 (18)	C9—N1—C8	124.65 (17)
C11—C10—C9	117.20 (18)	C9—N1—H1	120.6 (13)
C12—C11—C10	121.4 (2)	C8—N1—H1	114.6 (13)
C12—C11—H11	119.3	C24—N2—H2A	109.5
C10—C11—H11	119.3	C24—N2—H2B	109.5
C13—C12—C11	118.8 (2)	H2A—N2—H2B	109.5
C13—C12—H12	120.6	C24—N2—H2C	109.5
C11—C12—H12	120.6	H2A—N2—H2C	109.5
C12—C13—C14	121.8 (2)	H2B—N2—H2C	109.5
C12—C13—H13	119.1	C2—O1—C1	118.18 (19)
C14—C13—H13	119.1	C15—O2—C16	119.14 (17)
C13—C14—C15	119.4 (2)	C18—O3—C17	119.08 (19)
C13—C14—H14	120.3	H4B—O4—H4A	115 (3)
C15—C14—H14	120.3	Cl3—Zn1—Cl2	111.78 (2)
O2—C15—C14	123.99 (19)	Cl3—Zn1—Cl4	108.77 (3)
O2—C15—C10	115.83 (17)	Cl2—Zn1—Cl4	110.35 (3)
C14—C15—C10	120.17 (19)	Cl3—Zn1—Cl1	104.45 (3)
O2—C16—H16A	109.5	Cl2—Zn1—Cl1	111.19 (3)
O2—C16—H16B	109.5	Cl4—Zn1—Cl1	110.14 (3)
			
O1—C2—C3—C4	−178.8 (2)	C11—C10—C15—C14	−0.7 (3)
C7—C2—C3—C4	0.5 (3)	C9—C10—C15—C14	177.2 (2)
C2—C3—C4—C5	0.3 (4)	O3—C18—C19—C20	179.4 (2)
C3—C4—C5—C6	−1.0 (4)	C23—C18—C19—C20	0.5 (4)
C4—C5—C6—C7	0.9 (3)	C18—C19—C20—C21	0.8 (4)
C5—C6—C7—C2	−0.2 (3)	C19—C20—C21—C22	−0.9 (5)
C5—C6—C7—C8	179.77 (18)	C20—C21—C22—C23	−0.4 (4)
O1—C2—C7—C6	178.82 (17)	O3—C18—C23—C22	179.3 (2)
C3—C2—C7—C6	−0.5 (3)	C19—C18—C23—C22	−1.7 (4)
O1—C2—C7—C8	−1.1 (3)	O3—C18—C23—C24	−3.2 (3)
C3—C2—C7—C8	179.54 (18)	C19—C18—C23—C24	175.8 (2)
C6—C7—C8—N1	108.7 (2)	C21—C22—C23—C18	1.6 (4)
C2—C7—C8—N1	−71.3 (2)	C21—C22—C23—C24	−175.8 (2)
N1—C9—C10—C15	7.4 (3)	C18—C23—C24—N2	64.7 (3)
N1—C9—C10—C11	−174.7 (2)	C22—C23—C24—N2	−117.9 (2)
C15—C10—C11—C12	0.7 (3)	C10—C9—N1—C8	−174.76 (19)
C9—C10—C11—C12	−177.4 (2)	C7—C8—N1—C9	108.3 (2)
C10—C11—C12—C13	0.0 (3)	C3—C2—O1—C1	5.8 (3)
C11—C12—C13—C14	−0.8 (4)	C7—C2—O1—C1	−173.46 (18)
C12—C13—C14—C15	0.8 (4)	C14—C15—O2—C16	3.6 (3)
C13—C14—C15—O2	178.8 (2)	C10—C15—O2—C16	−177.5 (2)
C13—C14—C15—C10	−0.1 (3)	C23—C18—O3—C17	175.7 (2)
C11—C10—C15—O2	−179.57 (19)	C19—C18—O3—C17	−3.3 (4)
C9—C10—C15—O2	−1.7 (3)		

### Hydrogen-bond geometry (Å, °)

**Table:**

*D*—H···*A*	*D*—H	H···*A*	*D*···*A*	*D*—H···*A*
N1—H1···O2	0.89 (1)	2.07 (2)	2.680 (2)	125 (2)
N1—H1···Cl2^i^	0.89 (1)	2.64 (2)	3.3221 (18)	135 (2)
N2—H2A···O4	0.89	2.30	3.102 (3)	151
N2—H2A···O3	0.89	2.37	2.877 (2)	116
N2—H2B···O4^ii^	0.89	2.04	2.881 (3)	158
N2—H2B···Cl3^iii^	0.89	2.98	3.502 (2)	120
N2—H2C···Cl1^iv^	0.89	2.45	3.306 (2)	162
O4—H4B···Cl4^iii^	0.80 (2)	2.44 (2)	3.2323 (19)	168 (3)
O4—H4A···Cl1^v^	0.80 (2)	2.72 (2)	3.4165 (19)	147 (3)
C8—H8A···Cl3	0.97	2.67	3.474 (2)	140
C11—H11···Cl2	0.93	2.82	3.687 (2)	155
C24—H24A···Cl4^vi^	0.97	2.76	3.707 (3)	166

Symmetry codes: (i) *x*+1, *y*, *z*; (ii) −*x*+1, −*y*+1, −*z*; (iii) *x*, *y*, *z*−1; (iv) −*x*+1, −*y*+1, −*z*+1; (v) −*x*, −*y*+1, −*z*+1; (vi) *x*+1, *y*, *z*−1.
